# Assessing the Knowledge of Trauma Care Among Government Medical Officers in Uttarakhand: A Pre- and Post-intervention Study

**DOI:** 10.7759/cureus.92320

**Published:** 2025-09-14

**Authors:** Madhur Uniyal, Aditya Choudhary, Pushpendra Kaushik, Shantam Pokhriyal, Nidhi Kaeley, Neeraj Kumar, Ruby Kataria, Quamar Azam, Meenu Singh

**Affiliations:** 1 Trauma Surgery and Critical Care, All India Institute of Medical Sciences (AIIMS), Rishikesh, IND; 2 Emergency Medicine, All India Institute of Medical Sciences (AIIMS), Rishikesh, IND; 3 Trauma Surgery, All India Institute of Medical Sciences (AIIMS), Rishikesh, IND; 4 Pediatrics, All India Institute of Medical Sciences (AIIMS), Rishikesh, IND

**Keywords:** capacity building, chardham yatra, emergency medical services, medical training, trauma care

## Abstract

Background and objective: The Char Dham Yatra in Uttarakhand, India, attracts millions of pilgrims annually and poses a high risk of trauma in challenging high-altitude conditions. Trauma-related emergencies are a major concern in such regions, where healthcare resources are limited. Government medical officers, often deployed without formal trauma training, are crucial for timely care. Strengthening their knowledge through short, structured programs has wider implications for resource-constrained settings globally. This study evaluated a one-day trauma care training for Uttarakhand’s medical officers, aiming to enhance trauma management knowledge and inform strategies to improve emergency preparedness in similar high-risk environments.

Methods: This pre- and post-intervention study was conducted at All India Institute of Medical Sciences (AIIMS) Rishikesh (UK, IND) and included 125 government medical officers nominated by the Director General of Health Services, Uttarakhand. The training covered essential trauma care topics through faculty lectures, interactive sessions, and hands-on simulations. A validated 40-question pre-test and post-test were used to assess knowledge, while practical skills in airway management, breathing, hemorrhage control, neurological assessment, and patient transfer were evaluated using a five-point Likert scale. Paired t-tests were used to compare pre- and post-training scores.

Results: Post-training knowledge mean score significantly increased from 13.49 (pre-test) to 21.71 (post-test) (p < 0.001), marking a 61% improvement in trauma care knowledge. In skill assessments, 58.4% of participants were rated as outstanding or very satisfactory, 32.8% as satisfactory, and 8.8% as unsatisfactory, indicating areas requiring further training. These findings align with previous studies demonstrating the effectiveness of short-duration, structured trauma training programs in improving trauma management knowledge and skills. This study highlights the need for continuous skill reinforcement and refresher courses.

Conclusions: This study confirms that a one-day trauma training program significantly improves the knowledge and competence of medical officers in Uttarakhand, demonstrating its feasibility and impact in a resource-limited, high-demand environment. The model holds wider implications for India and other countries facing similar challenges of mass gatherings, remote geographies, and limited emergency infrastructure. Scalable training programs can strengthen healthcare capacity, potentially reducing morbidity and mortality from trauma across diverse settings.

## Introduction

Uttarakhand was established as the 27th state of India on November 9, 2000, after being carved out of northern Uttar Pradesh. Situated at the foothills of the Himalayan Mountain range, it is predominantly a hilly state with international borders shared with China (Tibet) to the north and Nepal to the east. Himachal Pradesh lies to its northwest, while Uttar Pradesh borders it to the south. The state is abundant in natural resources, particularly water and forests, and is home to glaciers, rivers, dense forests, and snow-covered mountain peaks. Uttarakhand, popularly known as *Devbhoomi*, or the 'Land of Gods,' is home to numerous temples and attracts devotees throughout the year. Among its many sacred circuits is the Char Dham Yatra, the most renowned pilgrimage, encompassing four Himalayan shrines, namely Yamunotri, Gangotri, Kedarnath, and Badrinath. In Hindi, *char* means 'four' and *dham* denotes 'holy abodes.' These high-altitude shrines remain closed during the winter months, opening in April or May and shutting again by October or November with the onset of snowfall [1]. The Char Dham Yatra, an annual pilgrimage, attracts three to five million pilgrims from across the globe [2]. The arduous trek, high altitude, unpredictable weather, and limited medical infrastructure contribute to increased morbidity and mortality among the pilgrims.

Road traffic incidents account for nearly 70% to 80% of trauma-related fatalities in India, disproportionately affecting young men, pedestrians, and cyclists. These injuries contribute to about one-third of all disabilities, with road traffic accidents responsible for nearly one-quarter of such cases. Beyond health impacts, the economic burden is substantial, as two-thirds of patients report out-of-pocket expenses ranging from ₹25,000 to ₹1,00,000, with a median of ₹40,000 [3]. India, despite having only 1% of the world’s vehicles, accounts for 11% of global road crash deaths, claiming about 40,000 young lives annually. In the past decade, crashes have caused 1.3 million deaths and over five million injuries, with an economic burden estimated at 3% to 5% of the nation’s GDP, according to the World Bank [4]. In 2021, India recorded 4,12,432 road accidents, leading to 1,53,972 fatalities and 3,84,448 injuries. The 18 to 45 age group was the most impacted, accounting for 67% of the deaths from these accidents [5]. A World Bank report notes that over 75% of low-income Indian households faced income losses after road crashes, averaging more than seven months’ earnings, compared to less than one month for wealthier families. Additionally, 64% reported a decline in living standards, and over 50% suffered post-crash depression, underscoring the disproportionate burden on vulnerable groups [6].

As per the Uttarakhand State Transport Department, in 2022, there were 1,674 reported accidents, with 851 of these resulting in fatalities and causing 1,042 deaths. The number of individuals severely injured in 2022 was 1,613, a notable increase compared to the previous year. It's important to note that these figures occurred despite COVID-related lockdowns and reduced public presence on the roads during those years [7]. Traumatic hemorrhagic shock has a high mortality rate, largely influenced by the severity and duration of reduced tissue perfusion, most of which occurs before hospital admission. The primary goal of Emergency Medical Services (EMS) in severe trauma cases is to minimize the time from injury to definitive care [8]. Upon arrival, a structured environment allows EMS to provide a concise patient report before departure. If necessary, the primary survey should begin during the handoff, ensuring a systematic assessment of airway, breathing, circulation, disability, and exposure (ABCDE) [9].

During the 2024 Char Dham Yatra, 183 deaths were reported within 100 days, including 177 from medical causes and six from natural disasters. The highest toll was at Kedarnath (83), followed by Badrinath (44), Yamunotri (31), Gangotri (15), and Hemkund Sahib (4) [10]. The influx of pilgrims during Char Dham Yatra places additional strain on the existing health services. Strengthening the primary and secondary health services not only benefits the local population but also ensures adequate medical support for the numerous pilgrims travelling the sacred paths of Uttarakhand. Such mortality, largely due to health-related emergencies in high-altitude, resource-limited settings, highlights the urgent need to strengthen the trauma and emergency care capacity of frontline medical officers. Importantly, the challenges observed in Uttarakhand are not unique; similar risks exist in other mass gatherings, disaster-prone regions, and remote terrains globally. Scalable, structured training programs can therefore serve as a model not only for India but also for other countries facing comparable public health challenges in high-risk environments.

Medical officers deployed along the Char Dham Yatra route serve as frontline responders, delivering timely care to pilgrims who may suffer traumatic injuries, acute medical emergencies, or worsening of pre-existing illnesses during the journey. The medical officers are often young professionals who may have limited experience in managing emergencies or trauma cases. Comprehensive training in trauma care will enhance their skills and confidence in handling diverse medical scenarios encountered during the pilgrimage and mitigate the impact of healthcare challenges in remote and challenging environments. Thus, this study seeks to assess the impact on knowledge via a capacity-building program for medical officers participating in the Char Dham Yatra.

## Materials and methods

This study was conducted at the Department of Trauma Surgery and Critical Care, All India Institute of Medical Sciences (AIIMS), Rishikesh, Uttarakhand, India, in the year 2024. The study period was six months. The training was designed for government medical officers (MBBS) of Uttarakhand, nominated by the state, and delivered by experienced faculty from the Department of Trauma Surgery of AIIMS.

**Figure 1:**
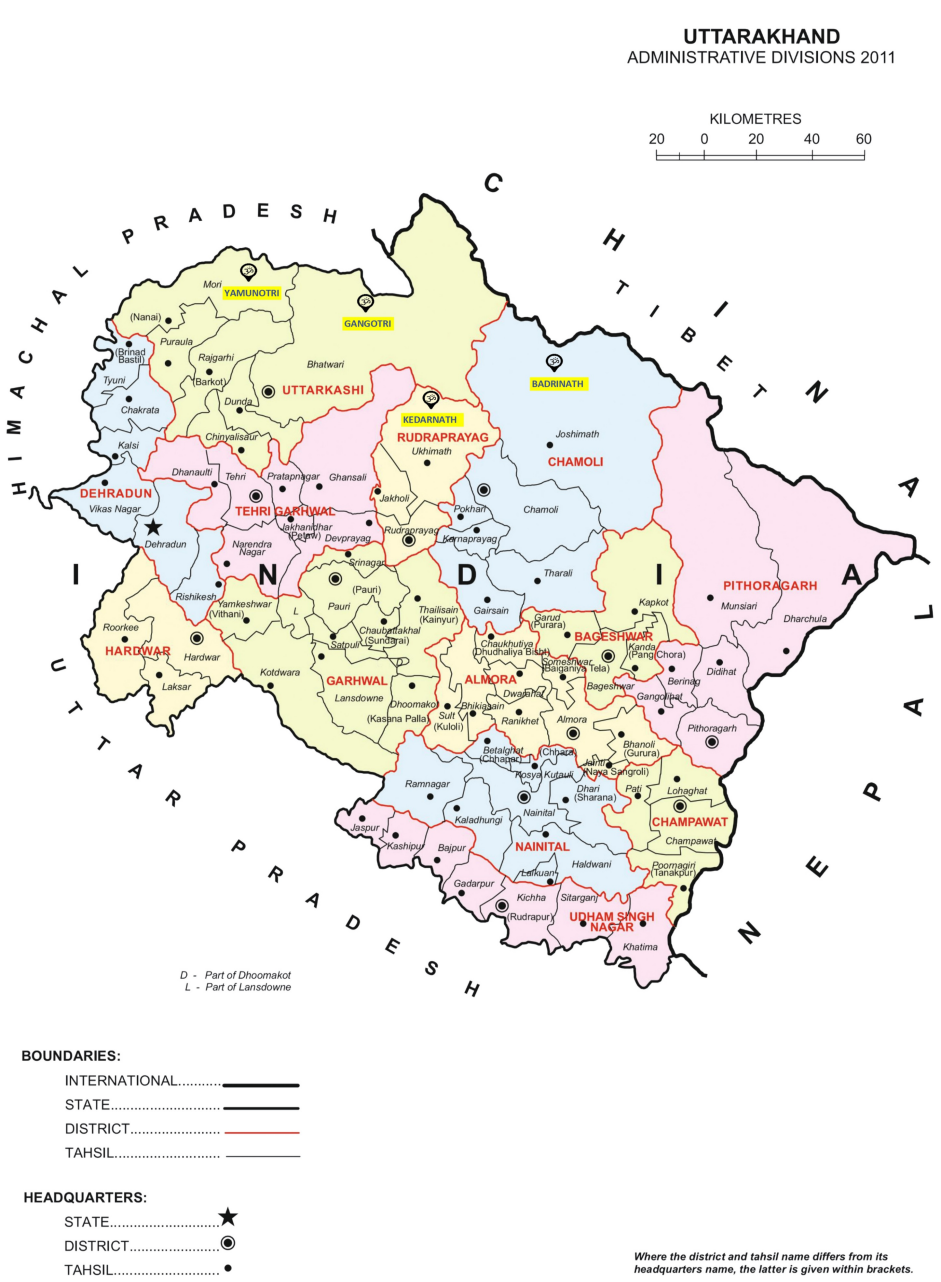
Map of Uttarakhand and its districts This map is sourced from the publicly available materials of the Office of the Registrar General & Census Commissioner, India (ORGI) Digital Library [11].

In total, 150 medical officers posted in the different districts of Uttarakhand (Figure 1) were nominated by the Director General of Health Services of the state, of whom 125 participated in the program. The faculty comprised experienced trauma surgeons from AIIMS Rishikesh and other medical colleges in Uttarakhand, with significant clinical and teaching expertise. They were selected based on their professional experience, active involvement in trauma care, and established contribution to trauma education.

The training followed standard trauma care guidelines and practices. Each session commenced with a pre-test comprising a validated 40-item questionnaire designed to assess participants’ baseline knowledge of trauma care. Trainings were conducted once weekly over six weeks and included didactic faculty lectures with slide presentations, supplemented by hands-on practical sessions focused on essential trauma care skills. The training protocol was developed in accordance with advanced trauma life support (ATLS) guidelines, simplified, and adapted to the specific requirements of Uttarakhand’s resource-limited settings. The sessions were standardized across all groups and supplemented by a structured training manual provided to participants for future reference. The topics covered and the hands-on skills practiced during the trainings are shown in Table 1.

**Table 1:** : Topics covered and the hands-on skills practiced during the trainings

Topics covered	Practical skills
Introduction and initial assessment	Airway
Airway and cervical spine	Breathing
Breathing and ventilation	
Circulation and haemorrhage control	Circulation
Disability	
Exposure and hypothermia prevention	Disability
Secondary survey and safe patient transfer	

Skill assessment was done through the skill assessment module at the simulation lab, AIIMS Rishikesh. This study employed an observational and evaluative approach to assess the competency of medical officers in performing critical trauma care tasks during the capacity-building program. A total of four trauma faculty members conducted the skill training. Each faculty member was assigned one specific skill station (airway, breathing, circulation, disability). To maintain uniformity, the faculty member who taught the skill also assessed participants’ performance at that station. This approach minimized inter-observer variability and ensured consistency in evaluation. Participants were evaluated on 10 specific skills, including helmet removal, primary and secondary surveys, hemorrhage control, airway and breathing assessment, Glasgow coma scale (GCS) scoring [12], and decision-making for patient transfer. Each skill was rated on a five-point Likert scale, ranging from 1 (poor) to 5 (outstanding), through direct observation by trained evaluators during simulated trauma scenarios. The evaluators, experienced healthcare professionals, used a standardized protocol adapted from ATLS guidelines to ensure consistency and objectivity in their assessments (Table 2). Post-training assessment was done after the training on the same day through a validated questionnaire comprising 40 questions. Changes in the technical scores of the participants were assessed.

**Table 2:** Assessment of competency in critical trauma care skills among healthcare providers 1 = Poor, 2 = Unsatisfactory, 3 = Satisfactory, 4 = Very satisfactory, 5= Outstanding

S. No	Skill	Scale
1.	Ability to show and able to perform helmet removal and c-spine movement restriction	1 2 3 4 5
2.	Performs the primary survey in trauma patients	1 2 3 4 5
3.	Demonstrated Team coordination in primary survey	1 2 3 4 5
4.	Ability to show techniques of haemorrhage control	1 2 3 4 5
5.	Performs the airway assessment in trauma patients	1 2 3 4 5
6.	Ability to diagnose breathing issues and understand the treatment modalities accordingly	1 2 3 4 5
7.	Assess the GCS Score (Glasgow Coma Scale) and focused neurological assessment	1 2 3 4 5
8.	Perform a methodical secondary survey of the trauma patients	1 2 3 4 5
9.	Utilise adjuncts of primary survey appropriately	1 2 3 4 5
10.	Appropriate decision-making for the transfer of the patients to definitive care	1 2 3 4 5

Before beginning the study, institutional permission was obtained from the Institutional Ethics Committee, AIIMS Rishikesh (approval no. AIIMS/IEC/24/172) after review of the study protocol. Informed consent was also obtained from the participants, and all the records were maintained with full confidentiality. Statistical analysis was performed using SPSS Statistics version 25 (IBM Corp., Armonk, NY, USA). Descriptive statistics, including proportions, percentages, means, and standard deviation (SD), were reported. For comparison of means between pre-test and post-test groups, a paired t-test was applied.

## Results

A total of 125 government medical officers participated in the training out of the 150 who were nominated by the state health department. Participants were from 11 districts of the state, but the distribution showed considerable variation. The majority of the participants were from Pauri Garhwal district (20%, n=125), followed by Tehri Garhwal (16%, n=125). The pattern of distribution of participants based on their place of posting is presented in Table 3 below.

**Table 3:** Pattern of distribution of participants based on their place of posting

District	N (%) (Total n = 125)
Pauri Garhwal	25 (20%)
Tehri Garhwal	20 (16%)
Almora	17 (13.6%)
Nainital	17 (13.6%)
Pithoragarh	13 (10.4%)
Chamoli	9 (7.2%)
Rudraprayag	9 (7.2%)
Uttarkashi	8 (6.4%)
Haridwar	4 (3.2%)
Dehradun	2 (1.6%)
Bageshwar	1 (0.8%)

For each correct answer, 1 mark was allotted; hence, the scores were out of 40 marks. The training proved to be effective overall, as evidenced by a notable increase in participants’ knowledge when comparing the pre- and post-training results. On average, there was an overall increase of 61% (n=125) in the knowledge of participants in trauma care when pre- and post-training results were compared.

There was a significant improvement from pre-test to post-test scores of the participants, as shown by the paired sample statistics. As seen in Table 4, the mean score in the pre-test was 13.49 ± 6.094 SD, which increased to 21.71 ± 4.327 in the post-test. The reduction in standard deviation and the smaller standard error in the post-test (0.387 compared to 0.545) indicate that the scores became more consistent and the mean estimate more precise. These results suggest that the intervention was effective in significantly enhancing participants' performance.

**Table 4:** Paired samples statistics for pre-test and post-test scores

Assessment	Mean	N	SD	Standard error (SE) mean
Pre-test	13.49	125	6.094	0.545
Post-test	21.71	125	4.327	0.387

Table 5 shows the paired sample t-test results for pre- and post-test scores. As seen in the table, the mean difference is -8.224 (SD = 4.140), which lies in the 95% confidence interval of -8.957 to -7.491. The t-statistic value is -22.207, and the p-value is <0.001. This demonstrates a significant effect of the intervention, affirming its effectiveness in improving participants' performance.

**Table 5:** Paired samples t-test results for pre-test and post-test scores

Assessment	Mean	SD	SE Mean	t	Significance (two-tailed)
Pre-test and post-test	-8.224	4.140	0.370	-22.207	0.000

The skill assessment results show that most participants demonstrated strong proficiency in trauma patient management, with 20% (n=125) rated as outstanding and 38.4% (n=125) as very satisfactory. A significant 32.8% (n=125) were assessed as satisfactory, indicating that the majority met the basic expectations (Table 6). However, 8.8% of participants were rated as unsatisfactory, highlighting a need for improvement in certain areas. Overall, the data suggests that while most participants possess solid skills, targeted training may be necessary for some to reach the desired competency level.

**Table 6:** Skill-wise performance distribution of participants (n = 125) 1= Poor, 2= Unsatisfactory, 3= Satisfactory, 4= Very satisfactory, 5= Outstanding GCS: Glasgow coma scale

Skill No.	Trauma Skill Assessed	Poor n (%)	Unsatisfactory n (%)	Satisfactory n (%)	Very satisfactory n (%)	Outstanding n (%)
1	Ability to show and perform helmet removal and c-spine movement restriction	4 (3.2%)	41 (32.8%)	35 (28.0%)	16 (12.8%)	29 (23.2%)
2	Performs the primary survey in trauma patients	0 (0.0%)	8 (6.4%)	30 (24.0%)	37 (29.6%)	50 (40.0%)
3	Demonstrated team coordination in primary survey	0 (0.0%)	12 (9.6%)	28 (22.4%)	37 (29.6%)	48 (38.4%)
4	Ability to show techniques of haemorrhage control	0 (0.0%)	17 (13.6%)	24 (19.2%)	34 (27.2%)	50 (40.0%)
5	Performs the airway assessment in trauma patients	0 (0.0%)	6 (4.8%)	30 (24.0%)	28 (22.4%)	61 (48.8%)
6	Ability to diagnose breathing issues and understand the treatment modalities accordingly	0 (0.0%)	13 (10.4%)	22 (17.6%)	33 (26.4%)	57 (45.6%)
7	Assess the GCS score and focused neurological assessment	0 (0.0%)	6 (4.8%)	38 (30.4%)	34 (27.2%)	47 (37.6%)
8	Perform a methodical secondary survey of the trauma patients	11 (8.8%)	37 (29.6%)	31 (24.8%)	18 (14.4%)	28 (22.4%)
9	Utilise adjuncts of primary survey appropriately	13 (10.4%)	39 (31.2%)	40 (32.0%)	7 (5.6%)	26 (20.8%)
10	Appropriate decision-making for the transfer of patients to definitive care	10 (8.0%)	43 (34.4%)	33 (26.4%)	13 (10.4%)	26 (20.8%)

## Discussion

This study highlights the effectiveness of targeted educational interventions in enhancing the knowledge of medical officers in trauma management. As the results of our study reveal, there is a significant increase in the knowledge of participants post-training compared to pre-training, indicating that the training program had a positive impact. Similar studies comparing the effectiveness of training programs in enhancing participants' knowledge have also yielded similar results. A study done at Khon Kaen University, Thailand, assessed the knowledge level and skill base in nurse anesthetists before and after training. The results show that after training, the knowledge and skills were significantly improved. The mean pre-training score of knowledge, which was 50.32, increased to 75.40 post-training [13]. This finding is similar to the current study, and the results are statistically significant (p < 0.001) in both studies. A similar study conducted by Kivlehan et al. evaluated the change in participants' emergency care knowledge following a five-day course and revealed that the participants' knowledge increased from 19 points pre-training to 22 points post-training on a 25-point scale. These findings are statistically significant (p < 0.001) and provide evidence for the positive impact of such training activities [14]. Another study conducted by Naidoo et al. compared knowledge changes among healthcare workers who participated in a training program, revealing similar results as the current study, although the specific skills and contexts differ. They found that the mean knowledge scores increased from 59.5% pre-training to 66.5% post-training, and the increase in knowledge is statistically significant (p < 0.001) [15]. The findings of our study are comparable to another study done by Tenner et al., conducted in Sub-Saharan Africa to assess the feasibility and utility of the course on Basic Emergency Care and knowledge transfer, which reported significant improvement (p < 0.05) in post-course test scores as compared to pre-course scores [16].

The skill assessment results indicate that the majority of participants demonstrated strong proficiency in trauma patient management, with 58.4% rated as either outstanding or very satisfactory. This suggests that such a training program may enhance trauma care skills. The significant portion of participants (32.8%) assessed as satisfactory further supports that the training met the basic competency requirements for trauma management. The finding that 8.8% of participants were rated as unsatisfactory presents an opportunity for further skill development. This highlights the potential for targeted interventions and continued training to help all participants reach the desired level of competency in trauma care. In challenging environments like the Char Dham Yatra, where medical officers may encounter high-pressure situations and complex trauma cases, additional support and training can further strengthen their preparedness and effectiveness.

The value of structured trauma training is underscored by recent evidence from India. In a mixed-methods cohort study, Kumar et al. evaluated the impact of ATLS training on patient outcomes and reported a significant reduction in preventable deaths (from 30% to 15%) and potentially preventable deaths (from 40% to 25%), following implementation of ATLS courses [17]. Systematic trauma training improves provider knowledge, confidence, and survival outcomes. The significant post-training knowledge gains in our study reinforce this evidence and highlight the need to expand structured trauma training across India, particularly in high-risk regions like Uttarakhand, where timely trauma care is critical. In the Sub-Himalayan region, nearly half of spine injuries are linked to falls from height, reflecting hazards of step-farming and unstable terrain, particularly for women in outdoor work. Older adults in hilly areas also face higher fall rates and fear of falling, leading to reduced mobility and activity. Uneven, sloped ground thus creates both physical and psychological risks. In this setting, enhancing trauma knowledge among Uttarakhand’s medical officers is crucial for timely recognition and management of fall-related and altitude-related injuries, helping reduce morbidity and mortality [18,19].

In India, trauma remains a leading cause of morbidity and mortality, yet it continues to be underrepresented in the medical curriculum. Strengthening undergraduate and postgraduate training through structured trauma competencies is therefore essential. This can be achieved by mapping trauma-specific skills within the competency-based curriculum, using simulation and skill-lab-based training (e.g., ATLS scenarios, airway management, bleeding control), and providing rotations or workshops in trauma and emergency units. In addition, incorporating regular assessments and student feedback can ensure sustained learning and iterative improvement. Such steps would not only prepare future physicians to manage trauma effectively but also help address a major public health gap [20,21]. This study's findings strongly indicate that the training program effectively increased the trauma management skills of the medical officers. The significant improvement in knowledge not only validates the training program’s success but also points to potential avenues for improving trauma care through focused training efforts.

This study has certain limitations. Skill assessment was conducted only once after training, so direct pre-post skill improvement could not be measured. The study lacked a control group, limiting comparison with untrained participants. In addition, outcomes were assessed immediately post-training, without long-term follow-up to evaluate retention. Despite these limitations, the findings provide valuable insight into the role of short, structured training programs in strengthening trauma care capacity.

## Conclusions

This study demonstrates the effectiveness of a one-day targeted training program in enhancing trauma care knowledge among government medical officers in Uttarakhand. Post-test scores showed a 61% average improvement compared to pre-test scores, underscoring the value of such structured educational interventions. By enabling medical officers to demonstrate core trauma care skills, the program strengthens emergency trauma services in remote and high-risk environments such as the Char Dham Yatra. Beyond immediate gains, this initiative highlights how targeted training can build long-term healthcare capacity and potentially reduce morbidity and mortality during mass gatherings. Future directions should include periodic refresher workshops, simulation-based drills, and long-term follow-up assessments to ensure sustained knowledge retention and skill application. Incorporating trauma training modules within undergraduate curricula and ensuring structured exposure to emergency and trauma units will further build capacity.
